# Exploration of Mechanisms of Sacubitril/Valsartan in the Treatment of Cardiac Arrhythmias Using a Network Pharmacology Approach

**DOI:** 10.3389/fcvm.2022.829484

**Published:** 2022-04-13

**Authors:** Yu Zhou, Shibao Rui, Shengxin Tang, Changlin Ju

**Affiliations:** ^1^Department of Emergency, The First Affiliated Hospital of Wannan Medical College, Wuhu, China; ^2^Department of Cardiology, The First Affiliated Hospital of Wannan Medical College, Wuhu, China

**Keywords:** sacubitril/valsartan, cardiac arrhythmia, network pharmacology, molecular mechanism, heart failure

## Abstract

Significant reductions in the incidence of cardiac arrhythmia (CA) and sudden cardiac death (SCD), along with amelioration of heart failure, have been reported for treatment with Sacubitril/valsartan (SV). However, its anti-arrhythmic mechanism remains unclear. The current study aims to explore the anti-arrhythmic molecular mechanism of SV. The direct protein targets (DPT) of SV were extracted from DrugBank. The protein-protein interaction (PPI) network of SV DPTs was constructed using STRING, and the indirect protein targets (IPTs) were also identified. A search for arrhythmia-related genes was conducted using GeneCards and the Comparative Toxicogenomics Database (CTD). The DTPs, ITPs, and arrhythmia-related genes from the two datasets were combined in a Venn diagram, and the overlapping genes were identified as core target genes. The Gene Ontology (GO) and Kyoto Encyclopedia of Genes and Genomes (KEGG) enrichment analyses identified the top 20 biological processes and signaling pathways related to disease and the therapeutic effects of SV. The renin-angiotensin system, adrenergic signaling in cardiomyocytes, and gap junction pathways are strongly implicated in the effects of SV on CA. In conclusion, our bioinformatics analyses provided evidence pertaining to the possible antiarrhythmic mechanisms of SV and may contribute to the development of novel drugs for CA.

## Introduction

Cardiac arrhythmia (CA) is a group of heterogeneous diseases that often leads to abnormal pulse formation and conduction due to the mutation of potassium and sodium channels (1). Almost all antiarrhythmic drugs that are used to treat CA have arrhythmogenic effects. Amiodarone, a broad-spectrum antiarrhythmic drug, has limited long-term suitability in patients with heart failure (HF) due to its extracardiac toxicity (2). Ideal antiarrhythmic drugs can prevent serious arrhythmias, effectively control the ectopic rhythm, restore or maintain sinus rhythm, and have no toxic effects on hemodynamics and vital organs (3). The search continues for better antiarrhythmic drugs.

Sacubitril/valsartan (SV), as an angiotensin receptor-neprilysin inhibitor (ARNI), positively improves the imbalance between the rein-angiotensin-aldosterone-system (RAAS) and natriuretic peptide systems (4), and decreases rehospitalization rates and cardiovascular mortality of patients with HF compared with enalapril (5). More surprisingly, SV decreases the incidence of ventricular tachycardia, ventricular fibrillation, implantable cardioverter-defibrillator discharge, and SCD in patients with HF (6–8). Recent studies have shown that inflammation, fibrosis, and autoimmune mechanisms may also lead to electrical remodeling or ion channel dysfunction and promote arrhythmia (9–11). Drugs that inhibit myocardial remodeling (12), such as ARNI, may play antiarrhythmic roles by reducing the formation of ectopic impulses and reentry from the upstream mechanism (13). However, the controversy around SV remains: it increases the incidence of ventricular arrhythmias, especially in patients with ischemic heart disease (14, 15), although after adjusting the outcomes, the cardiovascular death caused by etiologies of HF such as ischemic, non-ischemic, and hypertensive cardiomyopathies are similar (16, 17). But, in men with ejection fraction of <35%, patients who recently took SV may be more prone to ventricular arrhythmias (18). The data between the antiarrhythmic effect of SV and the etiology, gender differences, and age categories of HF are still limited (4). Therefore, the relationship between SV and CA deserves further study. The question of whether SV has a direct or indirect antiarrhythmic effect or has a pro-arrhythmic effect, as with other antiarrhythmic drugs, remains to be answered.

Network pharmacology is a vital tool to indicate interactions between drugs and organisms. Drug pleiotropy may be considered in the context of interactions between the regulatory network of the drug target and the disease’ gene product. Thus, network analyses give insights into multiple actions of drugs and their mechanisms (19). This approach has been used successfully to clarify the multi-targeted regulation of Traditional Chinese Medicine for treating disease (20).

The current study investigates the molecular mechanisms by which SV ameliorates CA using the approach of network pharmacology. The resulting bioinformatics data may assist with basic research and enable the development of new drugs for CA.

## Materials and Methods

### Recognition of Direct Protein Targets of Sacubitril/Valsartan

Drugbank is a network database containing comprehensive molecular information about drugs, their mechanisms, interactions, and targets. Since it was first released in 2006, Drugbank has been widely used to promote drug target discovery, design, docking, and screening and to predict metabolism and interactions in addition to general pharmaceutical education. A large number of new data have been added to DrugBank 5.0, including information on pharmacometabolomics, pharmacotranscriptomics, and pharmacoprotoemics, as well as hundreds of new drug clinical trials (21). The direct protein targets (DPTs) of SV were derived from DrugBank 5.0.

### Protein-Protein Interaction Network and Signaling Pathways for the Direct Protein Targets of Sacubitril/Valsartan

Search Tool for the Retrieval of Interacting Genes (STRING)^1^ is an online database for searching known protein-protein interaction (PPI) networks. Cytoscape is an open-source software project, which integrates biomolecular interaction networks with high-throughput expression data and other molecular states, producing a unified conceptual framework (22). The PPI network of the DPTs of SV was constructed using STRING. No more than 50 interactors on the first and second shells were set as the cut-off criteria with the highest confidence (score = 0.9). Highly relevant genes obtained were used as IPTs, and then, IPTs and DPTs were visualized by Cytoscape (v.3.8.2).

### Human Cardiac Arrhythmia-Related Genomic Data Sources

GeneCards^2^ is a searchable database that provides comprehensive and user-friendly information on all annotated and predicted human genes. Arrhythmia-related genes were searched within GeneCards and 4,317 results were obtained. The top 400 genes were selected as the research object according to the ‘‘correlation score’’ ranking. The Comparative Toxicogenomics Database (CTD)^3^ is a powerful and open database designed to promote an understanding of how environmental exposure affects human health. It provides artificially curated information on chemical–gene/protein interactions, chemical–disease, and gene-disease relationships. A total of 383,061 results were obtained by searching “disease and arrhythmia” within the CTD database. Repeated genes were removed according to the descending order of “inference score” and the top 400 genes were selected as research objects.

### Obtaining Potential Therapeutic Target Genes for Sacubitril/Valsartan Concerning Human Arrhythmia

The DPTs and IPTs of SV and CA-related genes from the GeneCards and CTD databases were introduced into a Venn diagram.^4^ Through integration and intersection, potential therapeutic target genes of SV for arrhythmia were obtained.

### Establishing Protein-Protein Interaction Network, Gene Ontology, and Kyoto Encyclopedia of Genes and Genomes Enrichment Analyses of Target Genes and Network Construction for Drug-Target Genes-Disease

Overlapping genes for the drug and disease were imported into the STRING database and a PPI network was constructed. The screening conditions were the species, “Homo sapiens” and a combined score of >0.4. A PPI diagram was constructed in which each node represented a gene and the nodes were connected by lines. The GO and KEGG were used to enrich and analyze overlapping genes using Omicshare tools,^5^ a free online data analysis platform.

**FIGURE 1:**
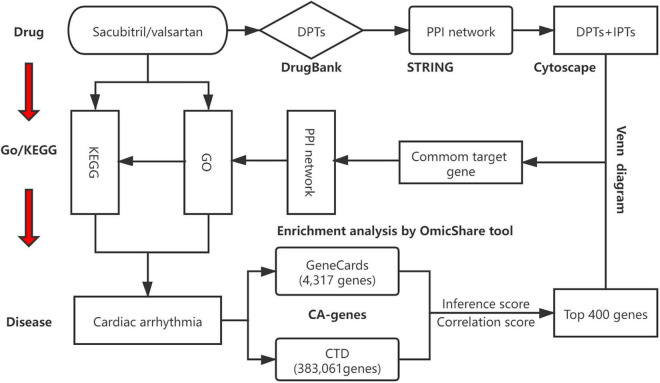
A schematic diagram showing the network pharmacological method for identifying targets, PPI network, biological processes, and key pathways of SV acting on cardiac arrhythmia (CA). All known targets of Sacubitril/valsartan (SV) and CA were obtained through online databases. Anti-arrhythmia targets of SV were identified. After constructing a protein-protein interaction (PPI) network and determining the potential targets of SV concerning CA, gene ontology (GO) and Kyoto Encyclopedia of Genes and Genomes (KEGG) enrichment analyses were carried out. Finally, the SV-GO-KEGG-CA network was generated. CTD: comparative toxicology genomics database.

Network relationships of core target-related GO/KEGG enrichment of SV against CA were generated *via* Cytoscape software (v.3.8.2). The study design is shown in Figure 1.

## Results

### Identification of Direct Protein Targets of Sacubitril/Valsartan

The SV was described as a neprilysin inhibitor and anti-hypertensive agent in output DB09292 and DB00177 from DrugBank5.0. We identified 6 DPTs of SV (Table 1). These were MME (Neprilysin), SLCO1B3 (Solute carrier organic anion transporter family member 1B3), SLCO1B1 (Solute carrier organic anion transporter family member 1B1), AGTR1 (Type-1 angiotensin II receptor), CYP2C9 (Cytochrome P450 family 2 subfamily C member 9), and ABCC2 (Canalicular multispecific organic anion transporter 1).

**TABLE 1:** Direct protein targets of sacubitril/valsartan in DrugBank.

Drugs	Gene Name	Uniprot ID	Functions	Actions
Valsartan	AGTR1	P30556	Target	Inhibitor
	CYP2C9	P11712	Enzyme	Substrate
	ABCC2	Q92887	Transporter	Substrate
	SLCO1B3	Q9NPD5	Transporter	Inhibitor
	SLCO1B1	Q9Y6L6	Transporter	Inhibitor
Sacubitril	MME	P08473	Target	Inhibitor

*DrugBank Accession Number Valsartan: DB00177, Sacubitril: DB09292.*

### Construction of a Protein-Protein Interaction Network for the Direct Protein Targets of Sacubitril/Valsartan and Further Searches for Closely Related Indirect Target Proteins

**FIGURE 2:**
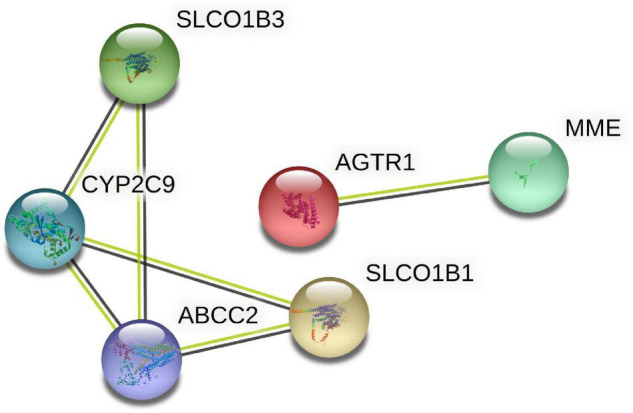
PPI network of the DPTs of SV constructed by STRING.

**FIGURE 3:**
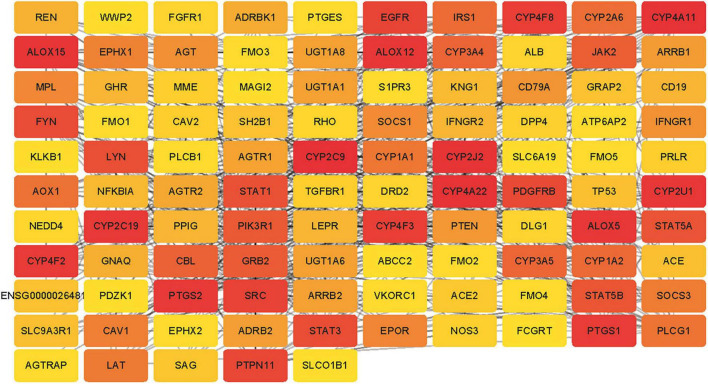
The PPI network for the DPTs and indirect protein targets (IPTs) of SV was constructed using STRING and no more than 50 interactors on the first and second shell were set as the cut-off criteria with the highest confidence (score = 0.9). The IPTs and DPTs were visualized by Cytoscape (v.3.8.2). The red to yellow rectangles represent scores from high to low.

Six target genes for SV were input into the STRING database. A PPI network was obtained after selecting “Homo sapiens” and a medium confidence of >0.4, as shown in Figure 2. There were 6 nodes and 6 edges in this network with a local clustering coefficient of 0.889. Conditions of not more than 50 interactors at 1st and 2nd shell, with the highest confidence of >0.9, were imposed and 105 DPTs and IPTs identified with 105 nodes and 493 edges (local clustering coefficient: 0.65; PPI enrichment *p* < 1 e-16) was entered into Cytoscape (3.8.2) to rebuild a PPI network of SV’s target genes, as shown in Figure 3.

### Identification of Target Genes Related to Cardiac Arrhythmia and Identification of Potential Therapeutic Target Genes for Sacubitril/Valsartan in Human Arrhythmia

**FIGURE 4:**
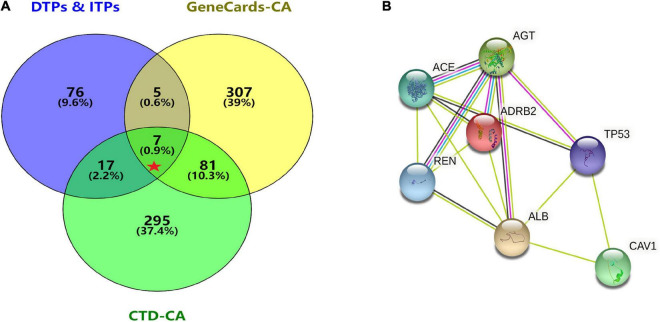
Acquisition of core target proteins and protein-protein interaction network. **(A)** SV-related drug target proteins [DTPs and indirect target proteins (ITPs)] were obtained from Drugbank Data and STRING. The top 400 CA-related genes were selected from GeneCards and CTD. The intersection of drug target protein genes (DTPs and ITPs) and the top two CA-related genes were selected by the Venn diagram. There were seven core target proteins (Red Pentagram). **(B)** A PPI network of the antiarrhythmic targets of SV was generated by the STRING database with medium confidence (0.400).

GeneCards were searched for CA-related genes and 4,317 results were obtained. The top 400 genes were selected as the research object according to the “correlation score” ranking. A search of “disease and arrhythmia” within the CTD database yielded 383,061 results. Repeated genes were removed according to the descending order of “inference score” and the top 400 genes were selected as the research object and analyzed using an online Venn diagram along with 105 DPTs and IPTs for SV. Seven core targets, ACE (Angiotensin I converting enzyme), CAV1 (Caveolin1), AGT (Angiotensinogen), REN (Renin), ADRB2 (Adrenoreceptor β2), TP53 (Tumor Protein P53), and ALB (Albumin) were identified from the intersections (Figure 4A).

### Establishing a Protein-Protein Interaction Network, Gene Ontology, and Kyoto Encyclopedia of Genes and Genomes Enrichment Analyses of Core Target Genes and Network of Drug-Target Genes-Disease

**FIGURE 5:**
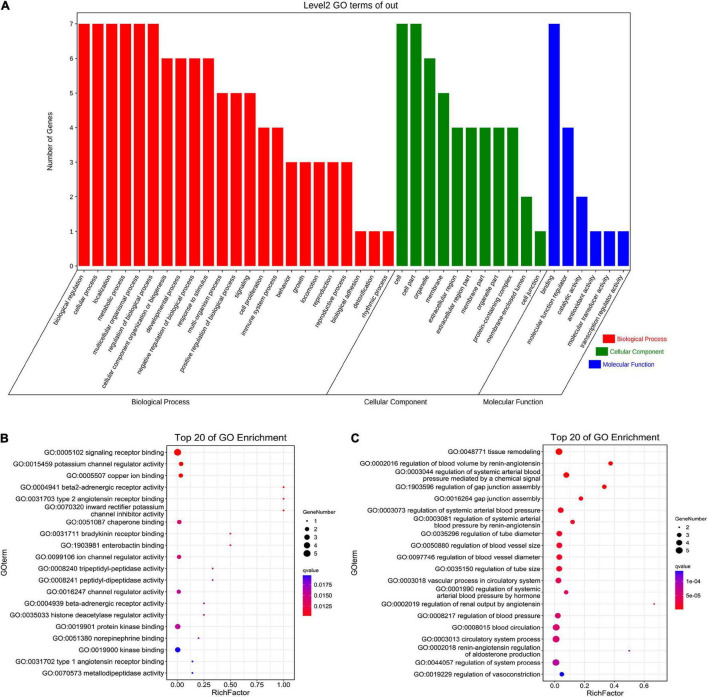
GO enrichment analysis of seven target genes (ACE, CAV1, AGT, REN, ADRB2, TP53, and ALB) for anti-arrhythmias with SV. **(A)** Biological process, cellular component and molecular function of Go enrichment of the target genes. **(B,C)** The bubble diagram shows the top 20 enriched molecular function and biological process respectively.

**FIGURE 6:**
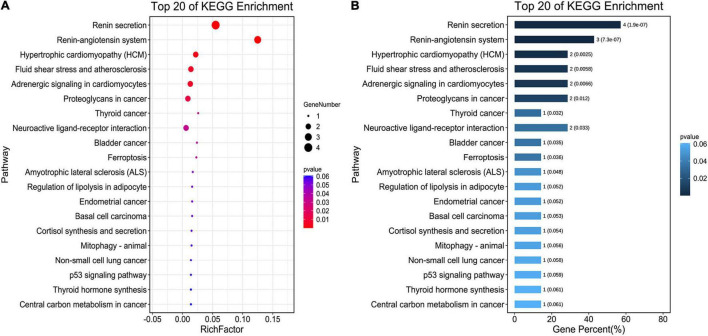
Pathway enrichment analyses of seven target genes (ACE, CAV1, AGT, REN, ADRB2, TP53, and ALB) for the treatment of CA with SV. **(A)** The bubble chart shows the top 20 enriched KEGG pathways. The *x*-axis indicates the Enrichment Factor and the intensity of different colors indicates the adjusted *p*-value. **(B)** The histogram shows the top 20 enriched KEGG pathways. The *x*-axis represents the enriched Gene Percentage (%) and the intensity of different colors indicates the adjusted *p*-value.

**FIGURE 7:**
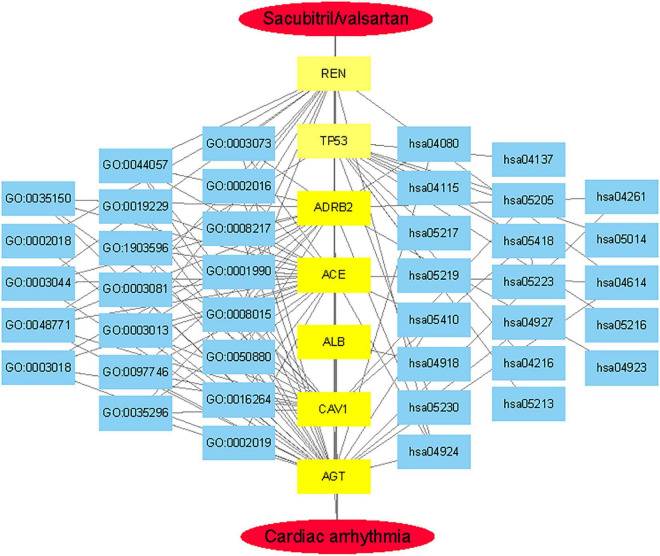
The integrated SV-GO-KEGG-Anti-arrhythmia network was visualized by Cytoscape.

The seven core target genes identified by the above method were imported into the STRING database and a PPI network was constructed (medium confidence: 0.400; Figure 4B). Overlapping genes were enriched and analyzed using GO and KEGG *via* OmicShare tools. Enriched molecular functions included signaling receptor binding, potassium channel regulator activity, copper ion binding, β2-adrenergic receptor activity, type 2 angiotensin receptor binding, and inward rectifier potassium channel inhibitor activity, among others (Figure 5A); Enriched biological processes included tissue remodeling, regulation of blood volume by renin-angiotensin, regulation of systemic arterial blood pressure mediated by a chemical signal, gap junction assembly, regulation of gap junction assembly, and regulation of systemic arterial blood pressure, among others (Figures 5B,C). The enriched molecular signaling pathways of the core targets were involved in renin secretion, renin-angiotensin system, hypertrophic cardiomyopathy, fluid shear stress, and atherosclerosis adrenergic signaling in cardiomyocytes, in addition to others (Figure 6). The network of the core targets for SV with therapeutic potential for CA and interaction diagrams of the core target related pathways were constructed (Figure 7).

## Discussion

The question addressed by the present study was whether SV has an antiarrhythmic or proarrhythmic effect in patients with HF. Using a network pharmacology approach, the current study reveals that SV, acting on core target genes (ACE, CAV1, AGT, REN, TP53, ADRB2, and ALB), has both direct and indirect effects during the treatment of CA.

The occurrence and maintenance of CA are usually mediated by ectopic activity and re-entry. The prolongation of the action potential/shortening of the effective refractory period and structural remodeling leads to slow and heterogeneous conduction, which provides the basis for reentrant arrhythmia. It was found that inhibiting the over-activation of RAAS reversed myocardial remodeling (23), reduced myocardial fibrosis and myocardial heterogeneous conduction. The formation of reentry and the occurrence of arrhythmia were both reduced (6, 7, 24), indicating an antiarrhythmic role. The current study showed that SV regulates ACE, CAV1, AGT, REN, and ADRB2 expression, inhibits tissue remodeling, and regulates vascular tension and volume by inhibiting the pathway of RAAS. In addition to myocardial remodeling, the remodeling of gap junctions is central to the generation and maintenance of arrhythmias. Gap junction remodeling leads to uncoupling of the myocardium, affects the conduction of the myocardial current, and generates arrhythmia. The SV may reduce the ventricular arrhythmias induced by myocardial hypertrophy by acting on the gap junction pathway (25, 26). Therefore, SV plays an indirect antiarrhythmic role by reversing myocardial and gap junction remodeling. Interestingly, angiotensin-converting enzyme inhibitor (ACEI) also reversed myocardial and gap remodeling, but did not reduce the incidence of SCD (27), suggesting that the antiarrhythmic effect of SV maybe also be related to neprilysin inhibition.

A decompensated state of HF is also represented by the over-activation of the SNS and is associated with poor prognosis, contributing to increased mortality risk. Neprilysin inhibition has been shown to activate the neuropeptide system, including that of atrial natriuretic peptide and brain natriuretic peptide, and synergistically inhibit SNS activity with valsartan (28). One pilot study showed that after taking SV for 2 months, the burst frequency and income of muscle sympathetic nerve activity decreased significantly, while heart rate and blood pressure did not change (29). It is by this strong antagonistic effect on the SNS that SV can reduce the incidence of SCD in patients with HF with reduced ejection fraction, an action that ACEI are unable to achieve (8). The β-adrenergic receptor antagonists are effective in reducing the damaging effects of increased SNS activity and produce significant mortality benefits for patients with HF. The ADRB2 gene encodes the β2-adrenergic receptor. Researchers have found that ADRB2 strongly increases localized RyR2-related cAMP levels during the over-activation of the SNS associated with cardiac hypertrophy or HF and increases ventricular arrhythmias (30). The ADRB2 also increases infiltration by pro-inflammatory macrophages, induces the production of IL-18, and sequentially stimulates the expression of connexin43 (Cx43) in fibroblasts in a paracrine manner, resulting in gap junction remodeling, myocardial fibrosis, and deterioration of cardiac function (31). The current findings that SV regulates ADRB2 expression suggest that the drug plays a direct antiarrhythmic role through pathways of hypertrophic cardiology and adrenergic signaling in cardiomyocytes.

Furthermore, the current study also found that SV regulates the levels of cav1 and TP53 and plays an antiarrhythmic role through the fluid shear stress and atherosclerosis pathway. Atrial fibrillation was associated with decreased CAV1 in right and left atria (*p* = 0.03); Loss of CAV1 leads to cSrc tyrosine kinase activation, gap junction remodeling, and ventricular arrhythmia, slowing left ventricular conduction velocity, and increasing ventricular arrhythmia inducibility (32). Further studies have shown that, when cardiac RAAS activity is enhanced, the dissociation of CAV1 from cSrc leads to cSrc activation, impaired gap junction function, and increased tendency toward ventricular arrhythmia and sudden cardiac death (33). These findings indicate that SV targets the regulation of CAV1 and reduces arrhythmia risk in heart disease associated with the activation of RAAS. Increased fibrosis and activated TP53 signaling were demonstrated in heart tissue from patients with dilated cardiomyopathy disease with ventricular tachycardia (34).

Any molecular mechanisms, where SV promotes arrhythmia, were not found in the current study. Clinical reports have indicated that SV can increase the incidence of ventricular arrhythmias in patients with HF (8, 14), but with short follow-up times and small sample sizes. Etiology, gender differences, and inclusion of high-risk patients may also affect the results (4). Further basic and clinical research into SV is needed to clarify whether the drug has proarrhythmic effects.

## Conclusion

In summary, a network pharmacological approach was used to demonstrate that SV acted on seven core target genes (ACE, CAV1, AGT, REN, TP53, ADRB2, and ALB) and reversed myocardial and gap junction remodeling improved the imbalance of RAAS, SNS, and neuropeptide system and had an indirect and direct antiarrhythmic effect. The most enriched pathways were renin secretion, renin-angiotensin system, hypertrophic cardiomyopathy, fluid shear stress and atherosclerosis, and adrenergic signaling in cardiomyocytes. The current study may further guide the basic and clinical investigations on the antiarrhythmic or proarrhythmic effects of SV on CA and contribute to the discovery of ideal antiarrhythmic drugs.

## Limitations

We acknowledge some limitations of the current study. Firstly, SV was decomposed into two separate components, Sacubitril and Valsartan, and the combination was not studied leading to some potential experimental deviations by comparison with the clinically administered formula. Secondly, targets for SV with potential antiarrhythmic effects were collected using public databases, which may lead to some inaccuracies. Thirdly, the core targets identified in this study require further validation *via* both basic scientific and clinical studies.

## Data Availability Statement

The datasets presented in this study can be found in online repositories. The names of the repository/repositories and accession number(s) can be found in the article/supplementary material.

## Author Contributions

CJ designed and performed the research. YZ wrote and revised the manuscript. SR and ST analyzed the public data and revised the manuscript. All authors read and approved the final manuscript.

## Conflict of Interest

The authors declare that the research was conducted in the absence of any commercial or financial relationships that could be construed as a potential conflict of interest.

## Publisher’s Note

All claims expressed in this article are solely those of the authors and do not necessarily represent those of their affiliated organizations, or those of the publisher, the editors and the reviewers. Any product that may be evaluated in this article, or claim that may be made by its manufacturer, is not guaranteed or endorsed by the publisher.

## Footnotes

1
https://string-db.org/cgi/input.pl

2
https://www.genecards.org/

3
http://ctdbase.org/

4
https://bioinfogp.cnb.csic.es/tools/venny/

5
www.omicshare.com/tools
